# Supplementary material for: The Presence of Hypoechoic Micronodules in Patients with Hashimoto′s Thyroiditis Increases the Risk of an Alarming Cytological Outcome
Source: J Clin Med. 2021 Feb 7;10(4):638. doi: 10.3390/jcm10040638 (PMC7915457; doi:10.3390/jcm10040638)

Table S1. Data on numbers and sizes of biopsied nodules and all nodules revealed in the thyroid in relation to the variant of the ultrasound pattern of the thyroid parenchyma (UP-HT)

**UP-HT variant**	**mean No of thyroid nodules in patient** *p*	**No/% of biopsied nodules**	**mean No of biopsied nodules in patient** *p*	**mean volume of biopsied nodules ±SD (cm³)** *p*	**No/% of biopsied nodules <1 cm** *p*
(a) hypoechoic, homogeneous echotexture	2.2±1.3 *NS*	50/6.6	1.2±0.5 *NS*	1.8±3.2 *NS*	7/14.0 *<0.005 vs f*
(b) hypoechoic, heterogeneous echotexture	2.4±1.5 *NS*	107/14.1	1.4±0.7 *NS*	1.2±1.6 *NS*	17/15.9 *<0.01 vs f* *<0.05 vs c*
(c) marked hypoechoic, heterogeneous echotexture	2.2±1.3 *NS*	212/28.0	1.3±0.6 *NS*	1.5±2.9 *NS*	18/8.5 *<0.05 vs b*
(d) heterogeneous echotexture with hyperechoic, fibrous septa	1.9±1.1 *NS*	40/5.3	1.3±0.5 *NS*	2.4±4.4 *NS*	7/17.5 *<0.05 vs f*
(e) multiple, discrete marked hypoechoic areas	1.8±1.1 *<0.001 vs f*	124/16.4	1.2±0.5 *NS*	1.2±1.6 *NS*	14/11.3 *NS*
(f) normoechoic pseudo-nodular areas	2.7±1.5 *<0.001 vs e*	191/25.2	1.6±0.8 *<0.05 vs g*	2.3±5.2 *NS*	10/5.2 *<0.005 vs a* *<0.01 vs b* *<0.05 vs d*
(g) echostructure similar to connective tissue	1.4±0.5 *NS*	6/0.8	1.2±0.4 *<0.05 vs f*	0.7±0.5 *NS*	1/16.7 *NS*
(h) thyroid with no signs of HT	2.4±1.2 *NS*	28/3.7	1.2±0.54 *NS*	2.1±2.3 *NS*	3/10.7 *NS*
Total	2.2±1.4	758/100.0	1.4±0.6	1.7±3.5	77/10.1

Table S2. Sizes of biopsied nodules in relation to the category of cytological outcome according to the Bethesda System for Reporting Thyroid Cytology (BSRTC)

	**category of BSRTC**							
	**I**	**II**	**III**			**IV**	**V**	**VI**
				FLUS	***AUS***			
**mean volume ± SD (cm^3)**	1.2±1.5	1.9±4.0	1.3±1.6	*1.4±1.6*	*0.9±0.8*	0.4±0.1	0.4±0.3	0.4±0.9
**No nodules**	128	527	94	*86*	*8*	2	4	3
**No/% of nodules <1 cm**	11/8.6	54/10.2	8/8.5	*7/8.1*	*1/12.5*	0/0.0	2/50.0	2/66.7

Figure S1-4. Examples ultrasonographic patterns of thyroid parenchyma in Hashimoto's thyroiditis: 1) hypoechoic, homogeneous/fine echotexture; 2) hypoechoic, heterogeneous/coarse echotexture; 3) marked hypoechoic, heterogeneous/coarse echotexture; 4) heterogeneous echotexture with hyperechoic, fibrous septa (A – transversal section; B – longitudinal section)

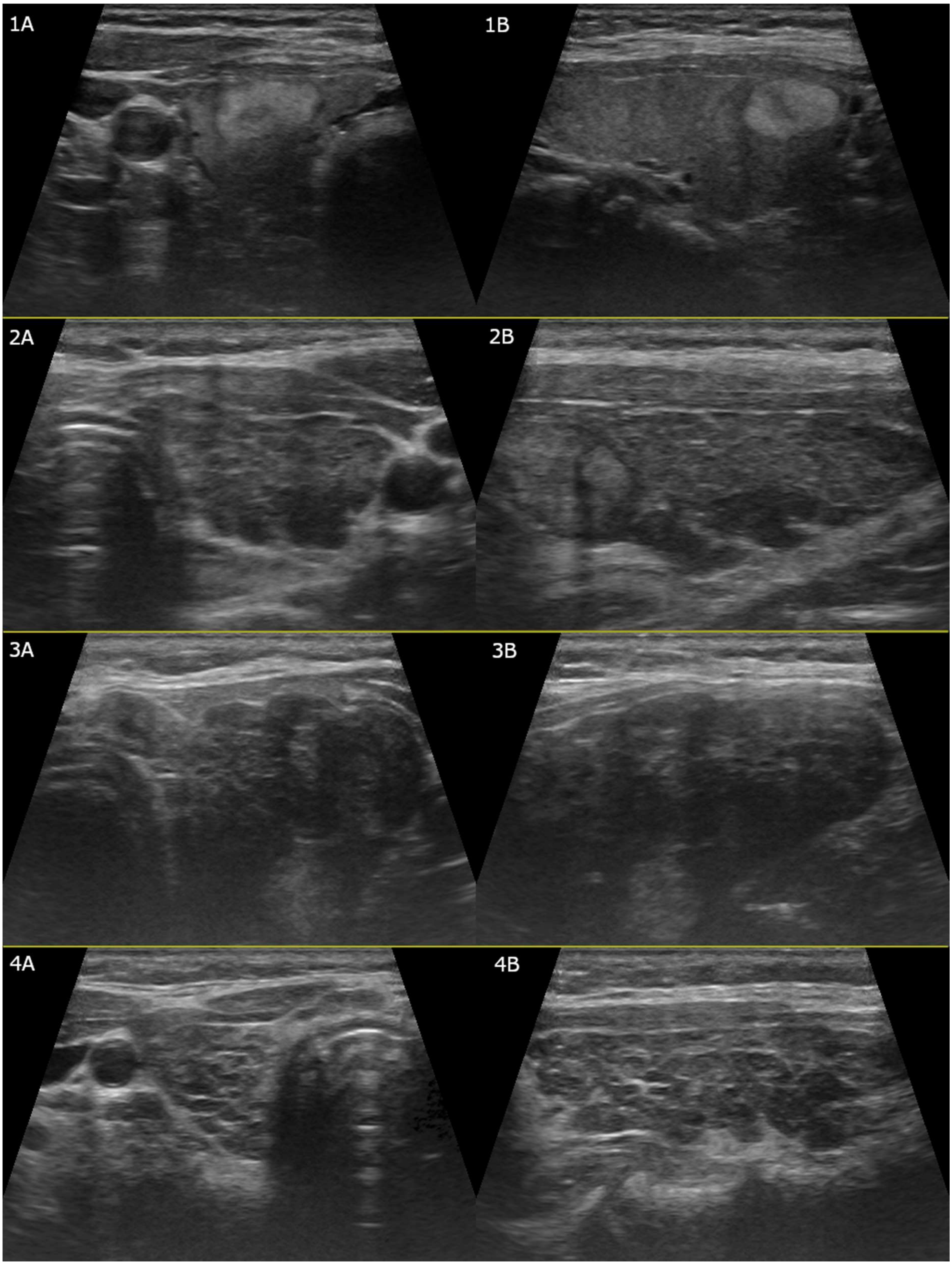

Figure S5-8. Examples ultrasonographic patterns of thyroid parenchyma in Hashimoto's thyroiditis (HT): 5) multiple, discrete marked hypoechoic areas, 6) normoechoic pseudo-nodular areas, 7) echostructure similar to connective tissue; 8) thyroid parenchyma with no signs of HT (A – transversal section; B – longitudinal section)

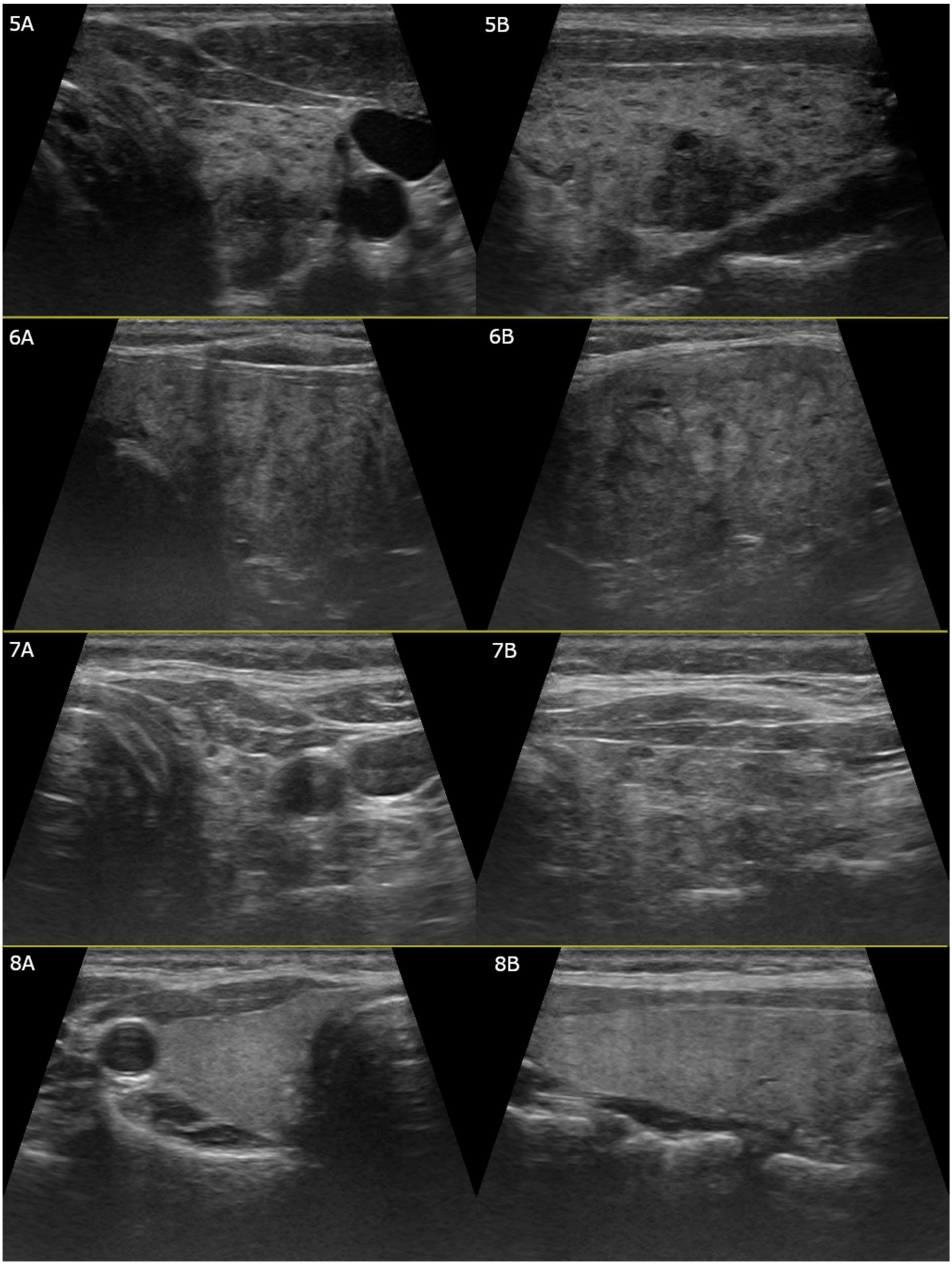

Supplement: Supplementary file 1 [file jcm-10-00638-s001.pdf]
